# Association of the rs1990760, rs3747517, and rs10930046 polymorphisms in the *IFIH1* gene with susceptibility to autoimmune diseases: a meta-analysis

**DOI:** 10.3389/fimmu.2023.1051247

**Published:** 2023-06-23

**Authors:** Zilin Xiao, Shuoming Luo, Yuemin Zhou, Haipeng Pang, Wenfeng Yin, Jiabi Qin, Zhiguo Xie, Zhiguang Zhou

**Affiliations:** ^1^ National Clinical Research Center for Metabolic Diseases, Key Laboratory of Diabetes Immunology (Central South University), Ministry of Education, and Department of Metabolism and Endocrinology, The Second Xiangya Hospital of Central South University, Changsha, Hunan, China; ^2^ Department of Epidemiology and Health Statistics, Xiangya School of Public Health, Central South University, Changsha, Hunan, China

**Keywords:** *IFIH1*, polymorphism, autoimmune diseases, type 1 diabetes, meta-analysis

## Abstract

**Objective:**

Interferon induced with helicase C domain 1 (*IFIH1*) single-nucleotide polymorphisms (SNP) rs1990760, rs3747517, and rs10930046 have been shown to be closely related to the risk of autoimmune diseases. The aim of this study was firstly to examine the association of the rs1990760 with type 1 diabetes (T1D) in a Chinese population. Secondly, to assess the association of SNP rs1990760, rs3747517, and rs10930046 with autoimmune diseases susceptibility.

**Methods:**

A total of 1,273 T1D patients and 1,010 healthy control subjects in a Chinese population were enrolled in this case-control study. Subsequently, we performed a meta-analysis on the association of the SNP rs1990760, rs3747517, and rs10930046 in the IFIH1 gene with susceptibility to autoimmune diseases. The random and fixed genetic effects models were used to evaluate the association and the effect sizes, including odds ratios (OR) and 95% confidence intervals (CI). Stratification analyses based on ethnicity and the type of autoimmune diseases were performed.

**Results:**

*IFIH1* SNP rs1990760 was not associated with a significant risk of T1D in the Chinese population in the case-control study. A total of 35 studies including 70,966 patients and 124,509 controls were identified and included in the meta-analysis. The results displayed significant associations between *IFIH1* rs1990760 A allele and rs3747517 C allele and autoimmune diseases risk (OR=1.09, 95% CI: 1.01~1.17; OR=1.24, 95% CI: 1.15~1.25, respectively). Stratified analysis indicated a significant association rs1990760 and rs3747517 with autoimmune diseases risk in the Caucasian population (OR=1.11, 95% CI: 1.02~1.20, OR=1.29, 95% CI: 1.18~1.41, respectively).

**Conclusions:**

This study revealed no association between *IFIH1* SNP rs1990760 and T1D in Chinese. Furthermore, the meta-analysis indicated that rs1990760 and rs3747517 polymorphisms, confer susceptibility to autoimmune diseases, especially in the Caucasian population.

## 1 Introduction

Autoimmune diseases (AIDs) are multifactorial diseases. Viral infections, genetic predisposition, and environmental factors contribute to the occurrence of AIDs (1, 2). Targeting different immune organs can lead to different AIDs, and sometimes multiple organs are involved. Common AIDs include type 1 diabetes (T1D), Grave’s disease (GD), Hashimoto’s thyroiditis (HT), systemic lupus erythematosus (SLE), rheumatoid arthritis (RA), multiple sclerosis (MS), and autoimmune Addison’s disease (AAD). Recent research has discovered that some AIDs may share several common mechanisms of pathogenesis or signaling pathways (3, 4), with some also sharing the same genetic background (5, 6). A previous genome-wide association study (GWAS) found that interferon-induced with helicase C domain 1 (*IFIH1*) rs1990760 was the third most associated single nucleotide polymorphism (SNP) with T1D (7, 8). Researchers also identified an association between several SNPs in the *IFIH1* gene with the risk of various AIDs, such as SLE (9), GD (10), and psoriatic arthritis (PsA) (11, 12).

*IFIH1*, also as known as melanoma differentiation-associated 5 (*MDA5*), participates in the innate immune system’s recognition of viruses and antiviral processes. The *IFIH1* gene, located at 2q24.3, encodes an apoptotic-associated protein activated by viral RNA, which acts as a positive regulator in the virus-induced activation of type I interferon (*IFN-I*) genes (13, 14). *IFIH1* can protects against viral challenges but modestly promotes the risk of autoimmunity (15). Extensive researches in different populations have examined the association of SNPs within the coding region of *IFIH1* with T1D (16–27). Previous a meta-analysis have confirmed the relationship between *IFIH1* SNP rs1990760 and T1D (28); however, few studies have been able to replicate this finding in T1D populations in China. Therefore, There is a need to revalidate the association between the *IFIH1* SNP rs1990760 in Chinese patients with T1D in a larger sample size.

Published articles on the association between common *IFIH1* gene SNPs, such as rs1990760, rs3747517, and rs10930046, and AIDs are constrained by various factors such as sample sizes, race, and/or clinical heterogeneity; as a result, contradictory or inconsistent conclusions were made (29). Therefore, to overcome the limitations of individual studies, and resolve inconsistencies, we conducted an all-sided meta-analysis of the association between three SNPs, including rs1990760, rs10930046, and rs3747517, in the *IFIH1* gene and multiple AIDs including SLE, T1D, GD, HT, RA, AAD, and MS. We included and retrieved the related case-control articles regarding the above seven autoimmune diseases published in recent years. In particular, the meta-analysis of the association between rs1990760 SNP with T1D included the present Chinese case-control study. The second objective in the present study was to assess the overall effect of the three *IFIH1* SNPs in AID susceptibility. To our knowledge, this is the most comprehensive meta-analysis investigating the impact of *IFIH1* SNPs on AID susceptibility at present.

## 2 Materials and methods

### 2.1 Subjects

This study consisted of two parts. Part one is the research data for the case-control study, and the second part is the literature retrieval data for the meta-analysis. In the case-control study for SNP rs1990760, a total of 1,273 T1D patients of Chinese Han origin (688 males and 585 females; average age at onset: 32.2 ± 17.4 years) were enrolled from October 1999 to December 2012. All participants were from the Department of Metabolism and Endocrinology, The Second Xiangya Hospital of Central South University. T1D was diagnosed based on the World Health Organization classification criteria of diabetes in 1999 (30). T1D patients were considered for inclusion as described previously (31, 32). A group of 1,010 control subjects was recruited from two cross-sectional surveys, including 614 males and 396 females, with an average age of 48.4 ± 14.1 years. The study met the international agreements of the World Medical Association Declaration of Helsinki 2000 regarding the ethical principles for medical research involving human subjects. The study protocol was approved by the ethical committee of The Second Xiangya Hospital of Central South University. Written informed consent was obtained from individual subjects.

DNA was extracted from whole blood by the phenol-chloroform method. Genotyping for rs1990760 was performed using TaqMan Assays in a 7900HT Fast Real-Time PCR system under the conditions recommended by the manufacturer (Array ID: C_2780299_30, Applied Biosystems, USA). An ABI Prism 7900HT Sequence Detection System and the SDS 2.2.2 software (both from Applied Biosystems) were used for allele discrimination. TaqMan typing results were verified by the direct sequencing.

The data retrieved from the literature were described below.

### 2.2 Publication search

Systematic literature searches (up to May 2022) were performed by using the online databases PubMed, Web of Science, and Embase, using the following search strategy: (“IFIH1” OR “MDA5”OR “rs1990760”, OR “rs3747517” OR “rs10930046”) AND (“gene” OR “Polymorphism” OR “genetic” OR “allele” OR “variant” OR “mutation”) AND (“autoimmune diseases” OR “autoimmune disease” OR “type 1 diabetes” OR “systemic lupus erythematosus” OR “Grave’s disease” OR “Hashimoto’s thyroiditis” OR “rheumatoid arthritis” OR “multiple sclerosis” OR “autoimmune Addison’s disease”). We also looked for other relevant studies via the references of all confirmed publications. Studies were included if they 1) investigated the association between *IFIH1* polymorphisms (rs1990760, rs3747517, or rs10930046) and AIDs; 2) were case-control studies; and 3) provided complete data to proceed with statistical analysis. Family-based association studies, conference abstracts, case reports, reviews, and comments were excluded.

### 2.3 Data extraction and quality assessment

We extracted the following data: the first author, year of publication, country, ethnicity of the participants, type of autoimmune diseases, number of genotypes in both patients and control groups, source of controls, and typing methods. In some studies (9, 17, 33–37), the genotypic data were obtained from the calculation of minor allele frequency (MAF), the total number of cases, and controls accordingly. Data extraction was done independently by Zilin Xiao and Yuemin Zhou. All differences were resolved by discussion. Data from each health cohort were evaluated for compliance with the Hardy-Weinberg equilibrium (HWE) using the *Chi*-square test. We used the Newcastle-Ottawa Scale (NOS) system to evaluate the quality of included case-control studies (overall NOS > 5) (38).

### 2.4 Statistical analysis and publication bias

The case-control association studies were analyzed using the *Chi*-square test on 2×2 and 2×3 contingency tables for allele and genotype frequencies, respectively. Odds ratios (ORs) and 95% confidence intervals (CIs) were calculated using *Woolf*’s method. Significance was defined by *P* < 0.05.

The whole process of the meta-analysis were conducted by STATA software (version 12.0, Stata Corporation, College Station, TX, USA). For rs1990760, rs3747517, and rs10930046, we performed homozygote (AA *vs.* GG; CC *vs.* TT; TT *vs* CC) and heterozygote (AA *vs.* AG; CC *vs* CT; TT *vs* CT) models, while the allele model (A *vs.* G; C *vs.* T; T *vs.* C), dominant model (AA + AG *vs.* GG; CC + CT *vs.* TT; TT + CT *vs.* TT), and recessive model (AA *vs.* AG + GG; CC *vs.* CT + TT; TT *vs.* CT + CC) were analyzed by the method of *Mantel-Haenszel* analysis. *P_HWE_* < 0.05 was considered statistically significant.

The pooled ORs and 95% CIs were used to assess the association between *IFIH1* SNPs and AIDs. The *Z*-test was used to examine the significance of the pooled ORs. *Higgins’ I^2^* statistic and the *Cochran’s Q* test were used to evaluate the heterogeneity of included studies. If *P_heterogeneity_* > 0.1 and I^2^ < 50.0%, heterogeneity among studies were excluded, and a favorable fixed-effects model was applied in the further meta-analysis; if not, the random-effects model was used. Subgroup analyses and sensitivity analyses were used to explore the source of heterogeneity. Regarding publication bias, a funnel plot was utilized for qualitative analysis, and *Begg’s* test and *Egger’s* test for quantitative analysis. In cases where *P_Egger_* < 0.05, the trim and fill method was used according to previous study. *Power* tests were performed using the G*Power software v.3.1.9.7. For the calculation of *Power* in our meta-analysis, the parameter effect size was converted from the corresponding OR value of each study according the previous studies (39).

## 3 Results

### 3.1 No association between the rs1990760 and T1D in the Chinese population

The clinical characteristics and laboratory data of patients with T1D showed in **Supplementary Table 1**. The genotype distributions from both T1D patients and controls were in HWE. To evaluate any associations between *IFIH1* rs1990760 and T1D, ORs for T1D in A/G allele and genotype, respectively, were calculated together with their corresponding 95% CI with the cohorts indicated in **Table 1** (α=0.05, power 100%). The results showed there was no significant association between the rs1990760 and T1D in the Chinese population (*P* > 0.05). Subsequent meta-analyses included this data. In addition, we divided the patients into two subgroups including the islet autoantibodies positive group and the negative group. However, no statistically significant association was found between these groups.

**Table 1:** Genotype and allele frequencies for rs1990760 in patients with T1D in Chinese association study.

	Genotypes (%)			Alleles (%)		*P* values		OR (95% CI) for A allele	Power^a^
	GG	AG	AA	G	A	Genotypes	Alleles		
Controls (n=1010)	657(65.0)	316(31.3)	37(3.7)	1630(80.7)	390(19.3)	>0.05	>0.05	1.04(0.89-1.20)	100%
T1D (n=1273)	812(63.8)	415(32.6)	46(3.6)	2039(80.1)	507(19.9)				
T1D with Ab+ (n=923)	590(63.9)	299(32.4)	34(3.7)	1479(80.1)	367(19.9)	>0.05	>0.05	1.04(0.88-1.21)	100%
T1D with Ab- (n=350)	222(63.4)	116(33.2)	12(3.4)	562(80.1)	140(19.9)			1.04(0.84-1.29)	100%

a using an α-error probability of 0.05 and the effect size to 0.3 (the medium) in the G*Power software.

### 3.2 Inclusion of studies in the meta-analysis

**Figure 1:**
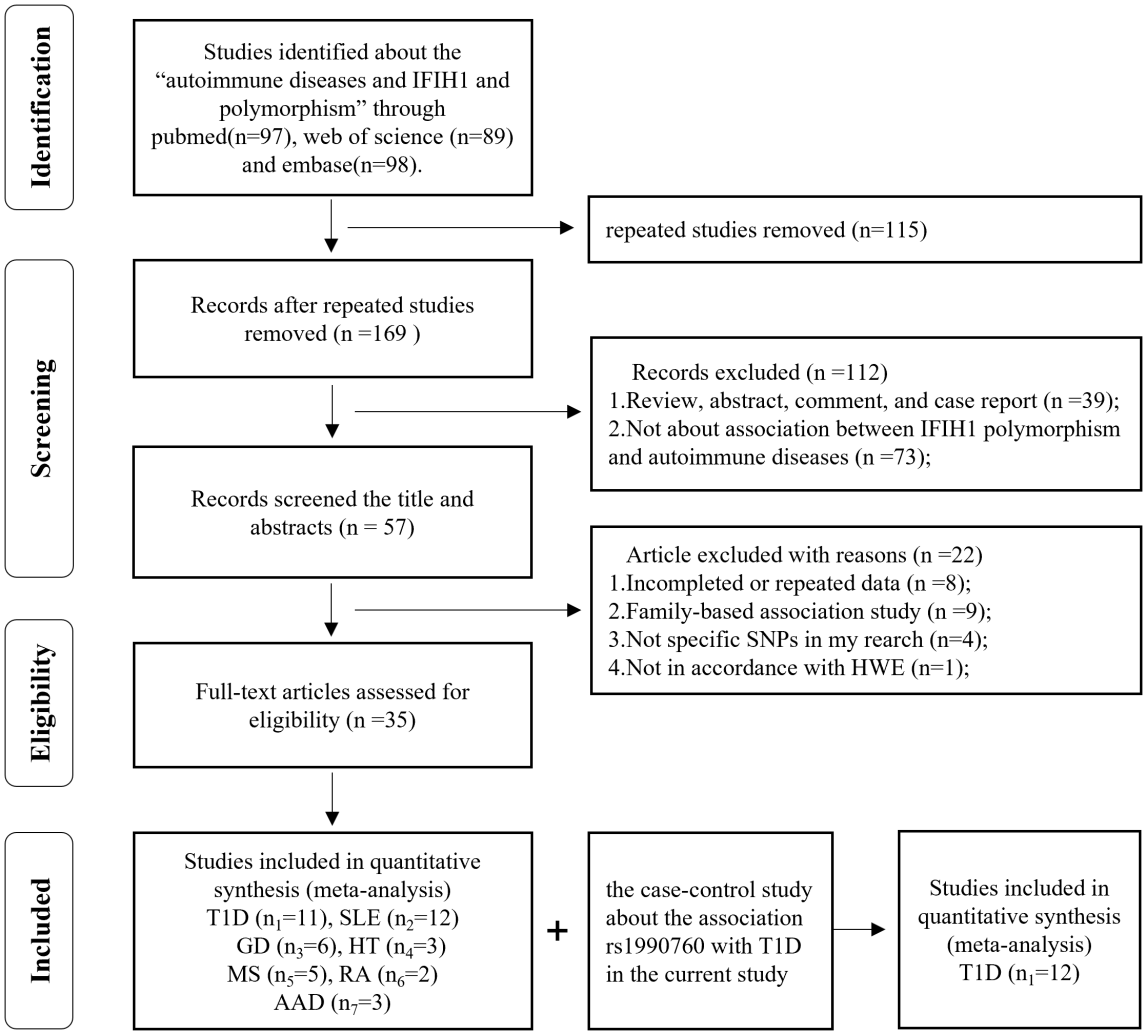
Flowchart for the identification of studies included in the meta-analysis.

We primarily searched a total of 284 potential articles from Pubmed (n=97), Web of Science (n=89), and Embase (n=98). We then excluded repeated studies (n=169). After screening the titles and abstracts, 39 articles were reviews, abstracts, comments, or case reports and 73 articles were not about the association between *IFIH1* polymorphisms and AIDs. Next, we read the full text and excluded eight studies with incomplete or repeated data. Nine studies were based on family, four studies included non-specific SNPs, and one study showed severe deviation from the HWE. Finally, 10 studies reported rs1990760, one study reported rs3747517, one study reported rs10930046, seven studies reported both rs1990760 and rs3747517, one study reported rs1990760 and rs10930046, and seven studies reported all three SNPs. The flow diagram of the study selection was shown in **Figure 1**.

Note that among the included studies, there were three multicenter case-control studies and seven studies including more than one autoimmune endocrine disease. One article’s genotype information was from a previous meta-analysis (40). Given the *IFIH1* rs3747517 polymorphism sample size, one study was selected (37), though it showed a slight deviation from HWE in controls (0.01 ≤ *P* < 0.05). One cohort was eliminated for deviating significantly (*P* < 0.05) from HWE in controls; however, we reserved another cohort in this article (19). People with one AID often had several AIDs simultaneously (21, 41). In an article that included both black and white participants, white participants were in the majority, thus, the data were categorized as white when racially stratified (20). In some studies, where subjects were divided into multiple subgroups, we included data from each subgroup as a separate study. Finally, 35 studies and 66 case-control comparisons were considered to meet the inclusion criteria, including eleven T1D studies (16–24, 29, 40), twelve SLE studies (9, 34–36, 42–49), eight GD studies (10, 24, 25, 50–53), three HT studies (51–53), five MS studies (16, 24, 33, 37, 54), two RA studies (55, 56), and three AAD studies (10, 41, 51), in total including 70,966 cases and 124,509 controls. The characteristics of the selected studies are summarized in **Table 2**.

**Table 2:** Characteristics of the included studies in the meta-analysis.

Author	Year	Ethnicity/Country	SNP	Disease		Cases			Controls		Source of control	Genotyping method	NOS	Power^d^
					11^a^	12	22	11^a^	12	22				
Martinez A	2008	Caucasian/Spain	rs1990760 A>G	T1D	120	151	40	188	254	93	Hospital	TaqMan	6	83.4%
				MS	170	183	62	188	254	93	Hospital	TaqMan		87.3%
Liu S	2009	Caucasian/US (Georgia)	rs1990760 A>G	T1D	638	637	159	680	892	293	Population	TaqMan	6	100%
			rs3747517 C>T	T1D	835	519	80	1008	726	131	Population	TaqMan		100%
		Caucasian/US(Denver)	rs1990760 A>G	T1D	243	285	84	204	263	85	Population	TaqMan		93.0%
			rs3747517 C>T	T1D	332	237	43	301	213	38	Population	TaqMan		93.0%
Nejentsev S	2009	Caucasian/UK	rs1990760 A>G	T1D	3280	3502	977	3789	4813	1573	Population	TaqMan	6	100%
			rs10930046 T>C	T1D	8159	195	2	10302	206	1	Population	TaqMan		100%
			rs3747517 C>T	T1D	4720	3120	539	5519	4216	840	Population	TaqMan		100%
Jermendy A	2010	Caucasian/Hungary	rs1990760 A>G	T1D	322	329	106	173	235	91	Population	TaqMan	6	94.6%
Yamashita H	2011	Asian/Japan	rs1990760 A>G	T1D	46	309	520	42	305	560	Unknown	TaqMan	6	98.9%
Yang H	2012	Asian/China	rs1990760 A>G	T1D	27	140	297	15	159	291	Hospital	TaqMan	7	86.7%
			rs3747517 C>T	T1D	88	262	114	57	198	210	Hospital	TaqMan		86.7%
Moura R	2013	Caucasian/Brazil	rs10930046 T>C	T1D	124	57	15	114	55	7	Population	TaqMan	6	49.3%
Bouças AP	2013	Caucasian/Brazil	rs1990760 A>G	T1D	150	263	114	139	239	139	Population	TaqMan	6	90.2%
Silva JA	2015	Caucasian/Brazil	rs3747517 C>T	T1D	63	53	9	77	76	17	Population	TaqMan	6	40.9%
			rs1990760 A>G	T1D	25	46	52	23	84	59	Population	TaqMan		40.2%
Zurawek M	2015	Caucasian/Poland	rs1990760 A>G	T1D	252	224	38	294	326	93	Population	TaqMan	6	94.2%
			rs3747517 C>T	T1D	346	156	12	386	269	58	Population	TaqMan		94.2%
Wawrusiewicz KN	2020	Caucasian/Poland	rs1990760 A>G	T1D	32	16	1	138	178	84	Population	TaqMan	6	56.9%
				MS	69	81	21	138	178	84	Population	TaqMan		67.3%
				GD	132	149	43	138	178	84	Population	TaqMan		77.3%
Ours	2022	Asian/China	rs1990760 A>G	T1D	46	415	812	37	316	657	Hospital	TaqMan	–	99.8%
Harley JB	2008	Caucasian/Multicenter	rs1990760 A>G	SLE	285	336	99	823	1128	387	population	Illumina Infinium HumanHap300	6	100%
Hom G	2008	Caucasian/Multicenter	rs1990760 A>G	SLE	533	605	172	2829	3773	1257	population	Illumina HumanHap550	6	100%
Gateva V	2009	Caucasian/US	rs1990760 A>G	SLE	431	533	165	1010	1456	525	population	Illumina Infinium II	6	100%
Gono T	2010	Asian/Japan	rs1990760 A>G	SLE	142	90	12	156	99	13	population	TaqMan	7	62.4%
Cunninghame G	2011	Caucasian/UK	rs1990760 A>G	SLE	343	388	114	2025	2589	835	population	Illumina	6	100%
Cen H	2013	Asian/China	rs1990760 A>G	SLE	46	297	534	35	306	637	population	Sequenom Mass ARRAY	7	99.1%
Molineros JE	2013	African/US (AA)^b^	rs1990760 A>G	SLE	974	490	61	3082	1272	131	population	Illumina iSelect	6	100%
			rs10930046 T>C	SLE	598	714	213	1467	2196	822	population	Illumina iSelect		100%
		Caucasian/US (EA)^b^	rs1990760 A>G	SLE	1550	1860	558	3510	4680	1560	population	Illumina iSelect		100%
			rs10930046 T>C	SLE	3842	125	1	9498	250	2	population	Illumina iSelect		100%
Wang C	2013	Caucasian/Sweden	rs1990760 A>G	SLE	467	525	148	767	980	313	population	Illumina BeadXpress	6	100%
			rs3747517 C>T	SLE	624	439	77	1039	848	173	population	Illumina BeadXpress		100%
Enevold C	2014	Caucasian/Denmark (CPH)^c^	rs1990760 A>G	SLE	52	74	16	229	305	107	Hospital	TaqMan	6	80.5%
		Caucasian/Denmark (ODE) ^c^	rs1990760 A>G	SLE	90	90	22	229	305	107	Hospital	TaqMan		83.3%
		Caucasian/Denmark(CPH)	rs3747517 C>T	SLE	73	61	8	311	279	51	Hospital	TaqMan		80.5%
		Caucasian/Denmark(CPH)	rs10930046 T>C	SLE	134	8	0	623	18	0	Hospital	TaqMan		80.5%
Silva JA	2016	Caucasian/Brazil	rs1990760 A>G	SLE	60	163	97	63	151	93	Hospital	TaqMan	6	76.5%
			rs3747517 C>T	SLE	171	135	25	158	132	22	Hospital	TaqMan		76.5%
			rs10930046 T>C	SLE	176	93	8	259	101	13	Hospital	TaqMan		76.5%
Zhang J	2018	Asian/China	rs1990760 A>G	SLE	17	143	236	21	191	447	Hospital	iMLDR	7	90.6%
			rs3747517 C>T	SLE	46	193	161	63	265	331	Hospital	iMLDR		90.6%
			rs10930046 T>C	SLE	298	98	4	512	140	7	Hospital	iMLDR		90.6%
Zedan MM	2021	Caucasian/Egypt	rs3747517 C>T	SLE	58	33	3	44	41	9	Hospital	TaqMan	7	28.2%
Sutherland A	2007	Caucasian/UK	rs1990760 A>G	GD	266	264	72	148	212	86	Population	mass spectrometry	7	90.3%
				AAD	69	105	30	148	212	86	Population	mass spectrometry		72.8%
Todd JA	2007	Caucasian/UK	rs1990760 A>G	GD	849	1042	293	1364	1764	577	Population	TaqMan	6	100%
Zhao ZF	2007	Asian/China	rs1990760 A>G	GD	16	75	171	16	75	171	Population	single-based primer extension technology	6	58.7%
Penna MM	2009	Caucasian/German	rs1990760 A>G	GD	88	132	38	87	112	28	Population	TaqMan	7	60.2%
				AAD	68	99	28	87	112	28	Population	TaqMan		54.4%
				HT	41	45	20	87	112	28	Population	TaqMan		45.2%
Ban Y	2010	Asian/Japan	rs1990760 A>G	GD	18	116	156	15	76	138	Population	LightScanner	6	63.1%
				HT	8	48	118	15	76	138	Population	LightScanner		52.5%
Rydzewska M	2018	Caucasian/Poland	rs1990760 A>G	GD	57	58	27	46	74	40	Population	TaqMan	6	41.7%
				HT	24	24	8	46	74	40	Population	TaqMan		31.7%
Enevold C	2009	Caucasian/Denmark	rs1990760 A>G	MS	372	468	123	336	462	162	Hospital	TaqMan	6	99.3%
			rs10930046 T>C	MS	0	37	926	0	25	935	Hospital	TaqMan		99.3%
			rs3747517 C>T	MS	533	375	55	466	419	75	Hospital	TaqMan		99.3%
IMSGC	2009	Caucasian/Belgium	rs1990760 A>G	MS	141	192	65	173	219	69	Hospital	TaqMan	6	83.9%
		Caucasian/Norway	rs1990760 A>G	MS	218	313	113	387	484	152	Hospital	TaqMan		98.4%
		Caucasian/UK	rs1990760 A>G	MS	862	1134	374	2608	3377	1093	Hospital	TaqMan		100%
Varzari A	2014	Caucasian/Germany	rs1990760 A>G	MS	101	336	279	110	337	258	Hospital	TaqMan	6	96.7%
			rs3747517 C>T	MS	396	273	47	370	282	54	Hospital	TaqMan		96.7%
Marinou I	2007	Caucasian/UK	rs1990760 A>G	RA	348	446	126	335	450	144	Population	TaqMan	6	99.1%
Martinez A	2008	Caucasian/Spain	rs1990760 A>G	RA	222	235	83	188	254	93	Hospital	TaqMan	6	91.0%
Zurawek M	2013	Caucasian/Polish	rs1990760 A>G	AAD	60	41	19	285	314	90	Population	TaqMan	6	81.7%
			rs3747517 C>T	AAD	74	39	7	372	261	56	Population	TaqMan		81.7%

a 1 and 2 represent the major allele and the minor allele, respectively, and the criteria for the major and minor alleles are based on the Caucasian population.

b AA means African Americans, EA means European-Americans.

c CPH cohort: from The Department of Rheumatology and Infectious Diseases, Rigshospitalet, Copenhagen; ODE cohort: from The Department of Rheumatology, Odense University Hospital.

d α=0.05, OR=1.2.

### 3.3 Association between *IFIH1* rs1990760 polymorphism and AIDs

The results of the meta-analysis for a possible association of the SNP rs1990760, rs10930046, and rs3747517 in the *IFIH1* gene with seven AIDs were summarized in **Table 3** and **Supplementary Table 2**.

**Table 3:** Meta-analysis about IFIH1 rs1990760, rs3747517 and rs10930046.

SNP	Genetic model	Stratification	Statistical model	Case/Control(n)^a^	OR (95%CI)	*P*(z test)^b^	I^2^	P_heterogeneity_ ^c^	P^d^
rs1990760	AA *vs.* GG	Overall	Random	39658/76434 (47)	1.26(1.17~1.36)	0.000	61.6%	0.000	100%
	AA *vs.* AG	Overall	Random	39658/76434 (47)	1.11(1.06~1.17)	0.000	45.3%	0.001	100%
	A *vs.* G	Overall	Random	39658/76434(47)	1.09(1.01~1.17)	0.021	91.5%	0.000	100%
	A *vs.* G	T1D	Random	15207/19054(12)	1.12(0.93~1.36)	>0.05	95.1%	0.000	100%
	A *vs.* G	SLE	Random	12863/38593(13)	1.08(0.93~1.25)	>0.05	94.7%	0.000	100%
	A *vs.* G	GD	Random	4062/5373(7)	1.16(1.00~1.34)	0.046	71.4%	0.000	100%
	A *vs.* G	HT	Random	337/616(3)	1.01(0.65~1.56)	>0.05	75.6%	0.016	5.3%
	A *vs.* G	MS	Random	5674/11163(7)	1.03(0.93~1.14)	>0.05	70.0%	0.003	56.3%
	A *vs.* G	RA	fixed	1505/1523(2)	1.12(1.00~1.24)	0.042	0.0%	0.406	93.1%
	A *vs.* G	AAD	fixed	519/1362(3)	1.05(0.90~1.22)	>0.05	0.0%	0.392	21.5%
		Caucasian	Random	33275/66998(37)	1.11(1.02~1.20)	0.017	92.9%	0.000	100%
		Asian	Random	4858/4951(9)	1.07(0.98~1.18)	>0.05	41.9%	0.088	96.0%
		African^e^	Random	1525/4485(1)	0.08(0.74~0.91)	0.000	–	-	100%
rs3747517	CC *vs.* TT	Overall	Random	15440/20522(14)	1.52(1.27~1.81)	0.000	59%	0.003	100%
	CC *vs.* CT	Overall	fixed	15440/20522(14)	1.16(1.11~1.22)	0.000	0.0%	0.606	100%
	C *vs.* T	Overall	Random	15440/20522(14)	1.24(1.15~1.25)	0.000	70.5%	0.000	100%
	C *vs.* T	T1D	Random	11528/14340(6)	1.30(1.11~1.52)	0.001	86.5%	0.000	100%
	C *vs.* T	SLE	fixed	2113/3827(5)	1.19(1.09~1.29)	0.000	28.2%	0.234	100%
	C *vs.* T	MS	fixed	1679/1666(2)	1.19(1.07~1.32)	0.002	16.6%	0.274	100%
	C *vs.* T	AAD	Random	120/689(1)	1.31(0.94~1.82)	>0.05	–	-	98.9%
	C *vs.* T	Caucasian	Random	14576/19398(12)	1.19(1.11~1.28)	0.000	55.7%	0.010	100%
	C *vs.* T	Asian	Random	864/1124(2)	1.52(1.14~2.04)	0.005	79.7%	0.026	100%
rs10930046	TT *vs.* CC	Overall	fixed	15868/27553(8)	1.48(1.24~1.74)	0.000	34.7%	0.176	100%
	TT *vs.* TC	Overall	Random	15868/27553(8)	0.88(0.72~1.09)	>0.05	77.2%	0.000	100%
	T *vs.* C	Overall	Random	15868/27553(8)	0.92(0.75 ~1.13)	>0.05	81.5%	0.000	100%
	T *vs.* C	T1D	fixed	8338/10509(2)	0.84(0.70 ~0.99)	0.039	0.0%	0.884	100%
	T *vs.* C	SLE	Random	6372/15908(5)	0.90(0.68 ~1.18)	>0.05	85.0%	0.000	100%
	T *vs.* C	MS	Random	963/960(1)	1.49(0.89 ~2.48)	>0.05	–	-	100%
	T *vs.* C	Caucasian	fixed	13943/22409(6)	0.85 (0.75 ~0.95)	0.006	22.3%	0.266	100%
	T *vs.* C	Asian	Random	400/659(1)	0.87(0.67 ~1.13)	0.000	–	-	65.4%
	T *vs.* C	African^e^	Random	1525/4485(1)	1.25 (1.15~1.36)	>0.05	–	-	100%

a n means the numbers of case–control cohorts.

b P(z test) <0.05 refuse H_0_: OR = 1 and statistically indicates a significant association.

c When I^2^ < 50% and P_heterogeneity_ > 0.1, enrolled data were considered with mild heterogeneity and the fixed-effect model was applicable.

d P means the power of the combined cohorts with α=0.05.

e African refers African Americans.

For the *IFIH1* rs1990760 polymorphism, 33 case-control studies containing 47 independent cohorts (39,658 cases and 76,434 controls) were included. The pooled ORs of all qualified case-control studies showed a significant association: allele model (A *vs.* G, OR=1.09, 95% CI: 1.01~1.17), dominant model (AA+AG *vs.* GG, OR=1.17, 95% CI: 1.10~1.24), recessive model (AA *vs.* AG+GG, OR=1.16, 95% CI: 1.10~1.22), homozygote (AA *vs.* GG, OR=1.26, 95% CI: 1.17~1.36), and heterozygote (AA *vs.* AG, OR=1.11, 95% CI: 1.06~1.17).

**Figure 2:**
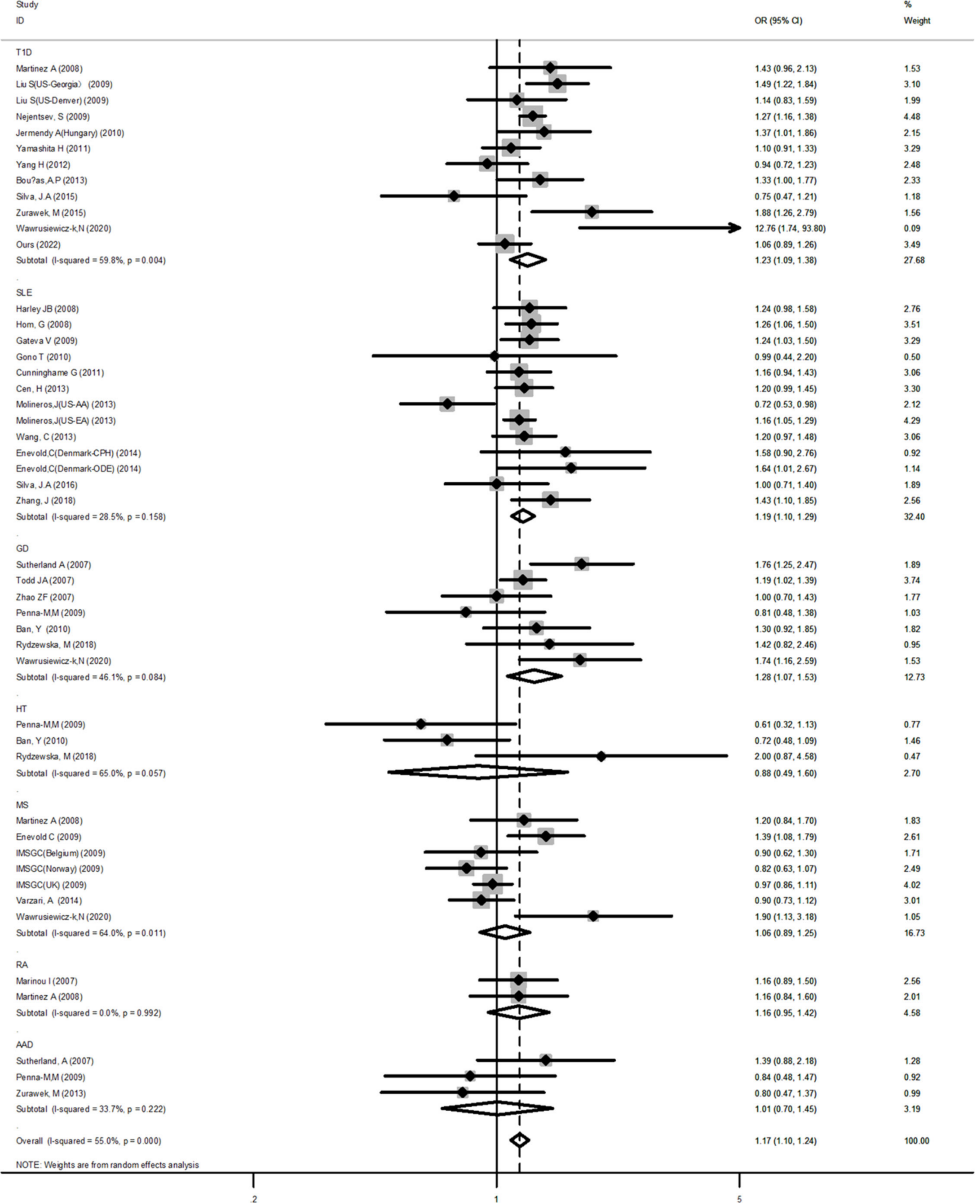
Forest plot for the meta-analysis of the association between the *IFIH1* rs1990760 polymorphism and diseases in the subgroup of autoimmune diseases. Synthesized data are based on 33 case-control studies containing 47 independent cohorts that reported *IFIH1* rs1990760 genotypic data, by the dominant model (AA+AG *vs* GG).

Subsequent subgroup analyses by diseases showed that among T1D, SLE, and GD, a significant association was observed in some genetic models. For example, in the dominant model (T1D: OR=1.23, 95% CI: 1.09~1.38; SLE: OR=1.19, 95% CI: 1.10~1.29; GD: OR=1.28, 95% CI: 1.07~1.53, respectively) (**Figure 2**). For AAD, no association was detected in any genetic models. But it is worth to mention that AAD association analysis was underpowered in all genetic models tested. RA showed a significant association only in the allele model (OR=1.12, 95% CI: 1.00~1.24). After stratifying by ethnicity, a significant positive association between AIDs and the *IFIH1* rs1990760 polymorphism was observed in the Caucasian population. However, for Asians, a significant association was observed only in the dominant model (OR=1.11, 95% CI: 1.02~1.20). The overall analysis suggested the existence of heterogeneity. Subgroup analyses reduced the heterogeneity for many diseases using the dominant and recessive models, but significant heterogeneity remained with the allelic model, even after subgroup analyses. Meanwhile, for HT and MS, the sensitivity analysis indicated a good stability for the estimated OR (**Supplementary Figure 1**). However, it should be pointed out that HT had a low power to detect a significant association depending on the model and MS was also underpowered for the recessive and allelic models.

### 3.4 Association between the *IFIH1* rs3747517 polymorphism and AIDs

For the *IFIH1* rs3747517 polymorphism, 13 case-control studies containing 14 independent cohorts (15,440 cases and 20,522 controls) were comprehensively compared. Due to the heterogeneity, all genetic models, except the heterozygote, used a random-effect model in the overall analysis. Results of the meta-analysis on associations between the *IFIH1* rs3747517 polymorphism and AIDs showed a significant association in the allele model (C *vs.* T, OR=1.24, 95% CI: 1.15~1.25), dominant model (CC+CT *vs.* TT, OR=1.40, 95% CI: 1.22~1.72), recessive model (CC *vs.* CT+TT, OR=1.23, 95% CI: 1.14~1.32), homozygote (CC *vs.* TT, OR=1.52, 95% CI: 1.27~1.81), and heterozygote (CC *vs.* CT, OR=1.16, 95% CI: 1.11~1.22).

**Figure 3:**
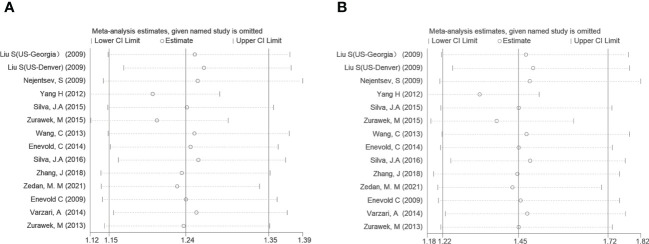
Sensitivity tests of *IFIH1* rs3747517 in the allele model **(A)** and dominant model **(B)**. Spots and horizontal line segments stand for estimated odds ratio (OR) values and 95% confidence intervals (CIs) of pooled data after omitting the labeled study in each line from the entire data pool. All estimated 95% CIs of OR ranges were above one, indicating a stable conclusion in the meta-analysis.

To further analyze the source of heterogeneity, subgroup analyses and sensitivity analyses were performed. After stratifying by diseases, T1D, SLE, and MS suggested a significant positive correlation (OR=1.30, 95% CI: 1.11~1.52; OR=1.19, 95% CI: 1.09~1.29; OR=1.19, 95% CI: 1.07~1.32; respectively, in the allele model). However, other AIDs did not show a statistically significant difference. Regarding ethnicity, both the Asian and Caucasian populations had significant associations in the above three models (OR=1.94, 95% CI: 1.16~3.24; OR=1.29, 95% CI: 1.18~1.41; respectively, in the dominant model). However, stratification by ethnicity could not explain the source of heterogeneity in the allele model and Asian in the dominant model. We performed a sensitivity analysis to evaluate the influence of the allele model and dominant model, which indicated a good stability for the estimated OR (**Figure 3**).

### 3.5 Association between the *IFIH1* rs10930046 polymorphism and AIDs

Seven research articles, including eight cohorts (15,868 cases and 27,553 controls), were enrolled. Pooled analysis detected a significant association in the dominant model (TT+TC *vs.* CC, OR=1.33, 95% CI: 1.15~1.55) and homozygote model (TT *vs.* CC, OR=1.48, 95% CI: 1.24~1.74) with the fixed-effect model. However, no association was observed among the allele model (T *vs.* C), heterozygote model (TT *vs.* TC), and the recessive model (TT *vs.* TC+CC) (all P>0.05).

There was no significant heterogeneity detected in the dominant model. The ethnicity subgroup analysis suggested inverse outcomes for Caucasians (OR=0.82, 95% CI: 0.72~0.92) and Africans (OR=1.33, 95% CI: 1.18~1.50) in the recessive model. Meanwhile, the problem of heterogeneity was solved. In the dominant model, only the African subgroup showed a statistical difference (OR=1.38, 95% CI: 1.17~1.63), and in the allele model, only the Caucasian subgroup showed statistical difference (OR=0.85, 95% CI: 0.75~0.95). The analysis stratified by the type of AID indicated that the SLE (OR=1.37, 95% CI: 1.17~1.60) subgroup and the T1D (OR=0.84, 95% CI: 0.70~0.99) subgroup had statistical significance. The results of the sensitivity analysis were shown in **Supplementary Figure 2**, which indicated a slight lack of stability for the estimated OR because of the limited sample sizes.

### 3.6 Publication bias

**Figure 4:**
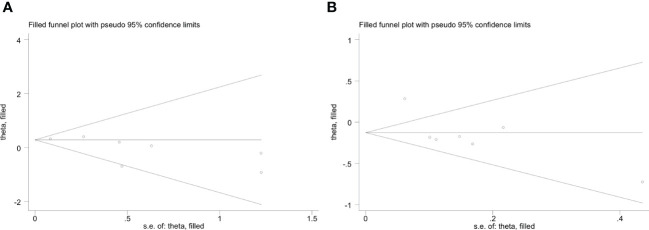
Trim and fill method funnel plots of *IFIH1* rs10930046 in the dominant model **(A)** and recessive model **(B)**. **(A)** Trim and fill method funnel plots of *IFIH1* rs10930046 in the dominant model. Symmetry was verified by Begg’s test with P_Begg’s_ = 0.035. **(B)** Trim and fill method funnel plots of *IFIH1* rs10930046 in the recessive model. Symmetry was verified by Egger’s test with P_Egger_ = 0.048.

The funnel plot of the *IFIH1* rs1990760 and rs3747517 polymorphisms indicated good symmetry, although rs10930046 did not. Publication bias was assessed using Begg’s test and Egger’s test (**Table 4**). *IFIH1* rs1990760 and rs3747517 showed no statistically significant bias under all models in both tests, while rs10930046 indicated statistically significant bias in different models (in the dominant model *P_Begg’s_* = 0.035; in the recessive model *P_Egger’s_* = 0.048). Therefore, we used a trim and fill approach to estimate the number of missing studies and incorporated missing hypothetical studies to recalculate the pooled OR values. However, no missing hypothetical studies were shown (**Figure 4**).

**Table 4:** The results of publication bias.

SNP	Begg’s test		Egger’s test	
	Z	P	t	P
rs1990760				
Allele model	0.37	>0.05	1.28	>0.05
Dominant model	0.13	>0.05	0.13	>0.05
Recessive model	0.26	>0.05	0.65	>0.05
rs3747517				
Allele model	1.53	>0.05	1.34	>0.05
Dominant model	0.77	>0.05	1.00	>0.05
Recessive model	0.88	>0.05	1.01	>0.05
rs10930046				
Allele model	0.37	>0.05	-2.37	>0.05
Dominant model	2.10	0.035	-2.00	>0.05
Recessive model	0.00	>0.05	-2.60	0.048

## 4 Discussion

As a gene closely related to the pathogenesis of AIDs, *IFIH1* has attracted much attention. Herein, we performed a risk association study between the *IFIH1* rs1990760 polymorphism and T1D in the Chinese population. Next, we carried out a comprehensive meta-analysis, including seven autoimmune diseases (T1D, SLE, GD, HT, RA, MS, and AAD), three SNPs of *IFIH1* (rs1990760, rs3747517, and rs10930046), and three ethnicities (Caucasian, Asian, and African), to investigate whether *IFIH1* polymorphisms were associated with AID susceptibility.

*HLA*, *INS*, and *PTPN22* are commonly demonstrated risk loci in T1D; another seven loci associated with a low polygenic risk score in T1D were identified in the updated GWAS (57). Recent GWAS and expression quantitative trait locus (eQTL) association studies have provided some new insights related to gene expression variation that may help to explain T1D susceptibility and phenotypic diversity (58). The GWAS, in addition to further verification in a case–control and family collection study, has shown evidence of the involvement of the *IFIH1* gene SNP rs1990760 in the risk of T1D (7, 59). Meta-analyses have also showed that *IFIH1* SNPs are related to T1D (20, 22), SLE (36), and PsA (60). Subsequent associations with *IFIH1* have been observed in other AIDs. The meta-analysis by Cen et al., which included literature published before 2013, estimated the association between AID risk and the *IFIH1* rs1990760 polymorphism, demonstrating that the *IFIH1* rs1990760 confers a risk of SLE, T1D, RA, and MS (45). Previous meta-analyses have mostly focused on one or two AIDs or just one polymorphism, whereas the present study aimed to comprehensively examine the possible association between three common *IFIH1* polymorphisms (rs1990760, rs3747517, and rs10930046) and the risk of seven common AIDs. In addition, we included studies published in recent years to ensure that we reported the latest developments.

In the current study, we did not find a significant association between the *IFIH1* SNP rs1990760 and T1D in a Chinese population. The results were consistent with two previous Asian studies (29, 40). The reason for this result may be the small population size, therefore, more studies based on Asian populations are needed in the future. The result of the current meta-analysis showed that the *IFIH1* rs1990760 allele A and its homozygote and heterozygote significantly increased the overall AID risk. The *IFIH1* SNP rs3747517 was also associated with AID susceptibility. Stratification by ethnicity and diseases could explain some of the heterogeneity. Begg’s test and Egger’s test showed no publication bias in rs1990760 and rs3747517. However, for *IFIH1* rs10930046, it was found to affect susceptibility to overall AID risk only in the homozygote and dominant model. Unexpectedly, there were some opposite susceptibility results within different disease types and between ethnic groups. This may be partly due to the lack of sufficient research and sample sizes from different sources in the subgroup analysis.

The subgroup analysis result of SNP rs1990760 suggested that it was associated with T1D, SLE, GD, and RA. However, we did not find a correlation between *IFIH1* and susceptibility to MS. These results differ from the results of Cen et al., which is probably because we included some newly published articles about GD and MS in our analysis. In addition, we indicated an association between rs1990760 and AID risk in Caucasians, whereas in Asians, only the dominant model showed significant statistical differences. The reason for the ethnic differences may be related to sunlight and other environmental factors, or it may be due to the insufficient sample size in the Asian studies. Rs1990760 and rs3747517 polymorphisms were associated with T1D by GWAS (7, 17, 18, 61); however, GWAS pays more attention to European populations. Therefore, a larger sample size is needed to investigate the association between *IFIH1* and AID susceptibility in Asian populations.

We cannot help but wonder if there is a common pathogenesis in these AIDs, and exploration of these mechanisms will help us to understand these diseases more deeply. Several recent studies have discussed this issue. One study analyzed two clinical trials showing the most common shared-targeting molecules and pathways, including the Janus kinase (JAK) signal transducer and transcriptional activator (STAT) pathways (4). The JAK-STAT signaling pathway revealed abnormal STAT signaling in inflammatory conditions and AIDs (62). *IFIH1* can augment IFN-α production (13) and the combination of IFN-α and IFNAR can animate the JAK-STAT pathway and induce the transcription of corresponding genes. Thus, *IFIH1* may induce the occurrence of AIDs through the JAK-STAT signaling pathway. One study has defined a common gene expression signature for some AIDs (63), and another has shown common genetic characteristics linked to AIDs (3). Meanwhile, *IFIH1* can activate the IFN-I response and pro-inflammatory cytokines after recognition of an enterovirus (64). Enterovirus infection may be associated with AIDs such as T1D (65).

There are some strengths and limitations to the current study. A strength is that our meta-analysis included 35 studies and 66 case–control comparisons, and we assessed seven AIDs and three *IFIH1* SNPs; therefore, it is the most comprehensive study of its kind to date. This could increase the statistical power of the overall analysis. However, we also note several study limitations. First, Caucasians constitute a large majority of the population in this study, which may lead to unavoidable geographical and ethnic bias. When performing the subgroup analyses, other races had far fewer cases and documents, thus the comparison may not be appropriate. Second, the number of relevant SNP rs10930046 studies was too small. If the meta-analysis included less than 20 original studies, the sensitivities of Begg’s test and Egger’s test were poor. rs1090046 has not shown significant differences in any single case–control study so far; therefore, statistical differences in a single model or stratification are not conclusive. Finally, we found that the original data included in the current meta-analysis were limited, so the influence of factors such as environment and population migration on gene and linkage disequilibrium remains unknown.

In conclusion, this meta-analysis showed that the *IFIH1* rs1990760 polymorphism A allele and *IFIH1* rs3747517 polymorphism C allele were associated with AIDs. Subsequent stratified analyses found that the rs1990760 polymorphism was associated with T1D, SLE, GD, and RA in different populations. SNP rs3747517 was associated with T1D, SLE, and MS. AIDs risk in both Caucasians and Asians was associated with the *IFIH1* rs3747517 polymorphism. Results from *IFIH1* rs10930046 did not reach universal statistical significance in our analysis.

## Data availability statement

The raw data supporting the conclusions of this article will be made available by the authors, without undue reservation.

## Ethics statement

The studies involving human participants were reviewed and approved by ethical committee of the Second Xiangya Hospital of Central South University. Written informed consent to participate in this study was provided by the participants’ legal guardian/next of kin.

## Author contributions

All authors contributed to the manuscript. ZLX wrote the first draft of the manuscript. SL and ZLX performed the material preparation. YZ and ZLX performed data collection. HP and WY contributed to part of the methods and tables. JQ and ZZ reviewed the manuscript and contributed to the discussion. SL proposed the project and is the guarantor of this work and, as such, had full access to all the data in the study and takes responsibility for the integrity of the data and the accuracy of the data analysis. All authors read and approved the final manuscript.
