# Supplementary figures and images for: Association of the rs1990760, rs3747517, and rs10930046 polymorphisms in the IFIH1 gene with susceptibility to autoimmune diseases: a meta-analysis
Source: Front Immunol. 2023 Jun 23;14:1051247. doi: 10.3389/fimmu.2023.1051247 (PMC10327432; doi:10.3389/fimmu.2023.1051247)

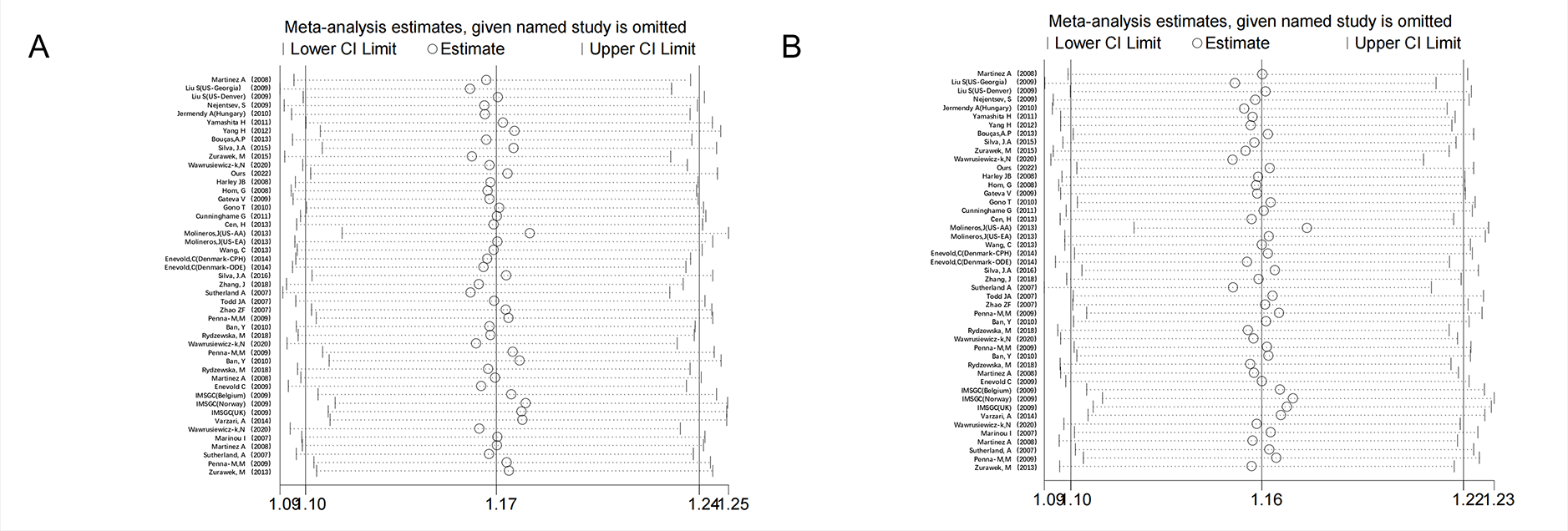

Supplement: Supplementary file 1 [file Image_1.tif]

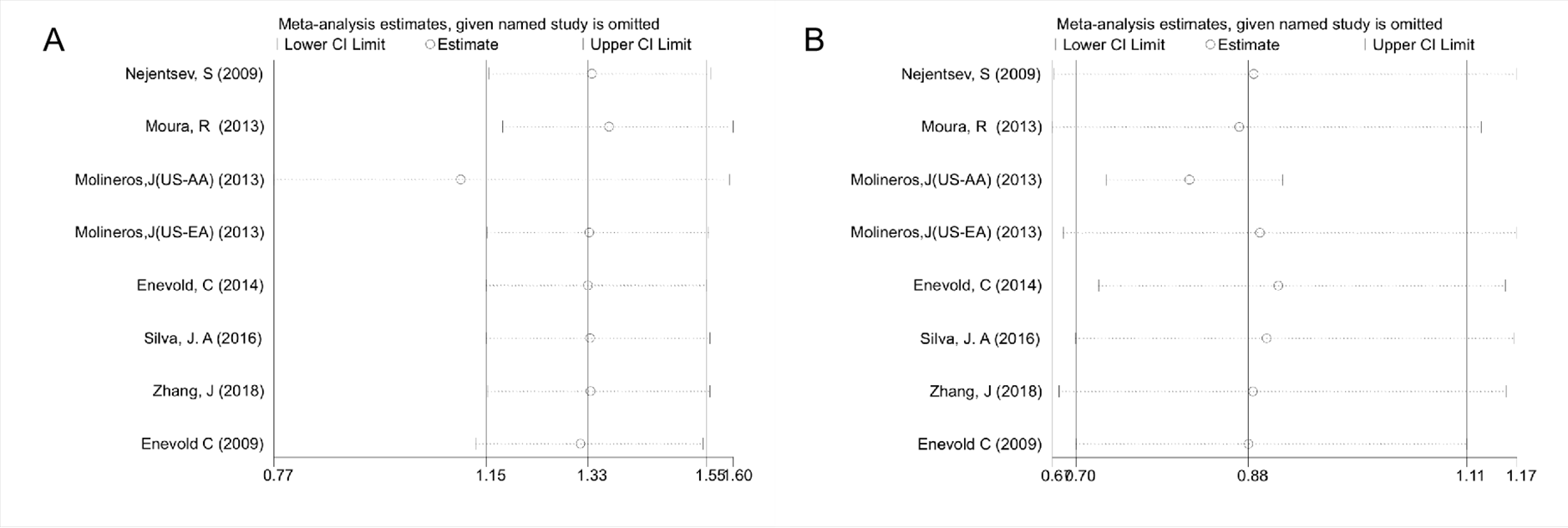

Supplement: Supplementary file 2 [file Image_2.tif]
