# Supplementary material for: Association of the rs1990760, rs3747517, and rs10930046 polymorphisms in the IFIH1 gene with susceptibility to autoimmune diseases: a meta-analysis
Source: Front Immunol. 2023 Jun 23;14:1051247. doi: 10.3389/fimmu.2023.1051247 (PMC10327432; doi:10.3389/fimmu.2023.1051247)
Supplement: Supplementary file 3 [file Table_1.docx]

**Supplemental Table 1** Clinical and laboratory characteristics of patients with T1D in the Chinese association study.

| Characteristics | Results |
| --- | --- |
| Male/Female | 688/585 |
| Age of diagnosis (years) | 32.2 ± 17.4 |
| Body mass index (kg/m^2^) | 20.7 ± 4.0 |
| HbA1c (%) | 10.1 ± 3.4 |
| GADA positive | 72.0% (780/1084) |
| IA-2A positive | 30.7% (267/869) |
| ZnT8A positive | 22.9% (141/615) |
| Susceptible DR3/DR3 frequency | 2.6% (28/1064) |
| Susceptible DR3/DR9 frequency | 4.7% (50/1064) |
| Susceptible DR9/DR9 frequency | 12.4% (132/1064) |
| Others non-susceptible genotype frequency | 80.3% (854/1064) |

Data are expressed as mean±SD, or percentage;

DR3, DRB1*0301-DQA1*05-DQB1*0201;

DR9, DRB1*0901-DQA1*03-DQB1*0303;
