# Supplementary material for: Association of the rs1990760, rs3747517, and rs10930046 polymorphisms in the IFIH1 gene with susceptibility to autoimmune diseases: a meta-analysis
Source: Front Immunol. 2023 Jun 23;14:1051247. doi: 10.3389/fimmu.2023.1051247 (PMC10327432; doi:10.3389/fimmu.2023.1051247)
Supplement: Supplementary file 4 [file Table_2.docx]

**Supplement Table 2. Meta-analysis about IFIH1** **rs1990760, rs3747517 and rs10930046 in Dominant model and Recessive model.**

| SNP | Genetic model | Stratification | Statistical model | Case/Control(n)^a^ | OR (95%CI) | *P*(z test)^b^ | I^2^ | P_heterogeneity_^c^ | P^d^ |
| --- | --- | --- | --- | --- | --- | --- | --- | --- | --- |

| rs1990760 | AA+AG *vs.* GG | Overall | Random | 39658/76434(47) | 1.17(1.10~1.24) | 0.000 | 54.9% | 0.000 | 100% |
| --- | --- | --- | --- | --- | --- | --- | --- | --- | --- |
|  | (Dominant model) | T1D | Random | 15207/19054(12) | 1.23(1.09~1.38) | 0.000 | 59.5% | 0.004 | 100% |
|  | AA+AG *vs.* GG | SLE | Fixed | 12863/38593(13) | 1.19(1.10~1.29) | 0.000 | 28.5% | 0.158 | 100% |
|  | AA+AG *vs.* GG | GD | Random | 4062/5373(7) | 1.28(1.07~1.53) | 0.006 | 46.1% | 0.084 | 100% |
|  | AA+AG *vs.* GG | HT | Random | 337/616(3) | 0.88(0.49~1.60) | >0.05 | 65.0% | 0.057 | 54.3% |
|  | AA+AG *vs.* GG | MS | Random | 5674/11163(7) | 1.06(0.89~1.25) | >0.05 | 64.0% | 0.011 | 98.7% |
|  | AA+AG *vs.* GG | RA | Fixed | 1505/1523(2) | 1.16(0.95~1.42) | >0.05 | 0.0% | 0.992 | 99.5% |
|  | AA+AG *vs.* GG | AAD | Fixed | 519/1362(3) | 1.01(0.70~1.45) | >0.05 | 33.7% | 0.222 | 5.7% |
|  | AA+AG *vs.* GG | Caucasian | Random | 33275/66998(37) | 1.20(1.12~1.29) | 0.000 | 54.2% | 0.000 | 100% |
|  | AA+AG *vs.* GG | Asian | Fixed | 4858/4951(9) | 1.11(1.02~1.20) | 0.017 | 30.3% | 0.176 | 100% |
|  | AA+AG *vs.* GG | African^e^ | Random | 1525/4485(1) | 0.72(0.53~0.94) | 0.039 | - | - | 100% |
|  | AA *vs.* AG+GG | Overall | Random | 39658/76434(47) | 1.16(1.10~1.22) | 0.000 | 62.9% | 0.000 | 100% |
|  | (Recessive model) | T1D | Random | 15207/19054(12) | 1.30(1.18~1.44) | 0.000 | 46.5% | 0.038 | 100% |
|  | AA *vs.* AG+GG | SLE | Random | 12863/38593(13) | 1.13(1.03~1.24) | 0.012 | 69.1% | 0.000 | 100% |
|  | AA *vs.* AG+GG | GD | Random | 4062/5373(7) | 1.20(0.10~1.45) | >0.05 | 55.5% | 0.036 | 100% |
|  | AA *vs.* AG+GG | HT | Fixed | 337/616(3) | 1.14(0.81~1.61) | >0.05 | 47.4% | 0.149 | 60.8% |
|  | AA *vs.* AG+GG | MS | Random | 5674/11163(7) | 1.02(0.91~1.14) | >0.05 | 48.9% | 0.068 | 29.5% |
|  | AA *vs.* AG+GG | RA | Fixed | 1505/1523(2) | 1.15(0.99~1.34) | >0.05 | 20.4% | 0.262 | 98.9% |
|  | AA *vs.* AG+GG | AAD | Fixed | 519/1362(3) | 1.08(0.87~1.34) | >0.05 | 37.3% | 0.203 | 45.4% |
|  | AA *vs.* AG+GG | Caucasian | Random | 33275/66998(37) | 1.17(1.11~1.23) | 0.000 | 56.3% | 0.000 | 100% |
|  | AA *vs.* AG+GG | Asian | fixed | 4858/4951(9) | 1.17(0.10~1.40) | >0.05 | 0.0% | 0.476 | 100% |
|  | AA *vs.* AG+GG | African^e^ | Random | 1525/4485(1) | 0.81(0.71~0.91) | 0.000 | - | - | 100% |
| rs3747517 | CC+CT *vs.* TT | Overall | Random | 15440/20522(14) | 1.45(1.22~1.72) | 0.000 | 65.1% | 0.000 | 100% |
|  | (Dominant model) | T1D | Random | 11528/14340(6) | 1.62(1.15~2.27) | 0.006 | 84.7% | 0.000 | 100% |
|  | CC+CT *vs.* TT | SLE | fixed | 2113/3827(5) | 1.37(1.15~1.62) | 0.000 | 0.20% | 0.405 | 100% |
|  | CC+CT *vs.* TT | MS | fixed | 1679/1666(2) | 1.30(0.99~1.70) | >0.05 | 0.0% | 0.536 | 100% |
|  | CC+CT *vs.* TT | AAD | Random | 120/689(1) | 1.43(0.64~3.21) | >0.05 | - | - | 100% |
|  | CC+CT *vs.* TT | Caucasian | fixed | 14576/19398(12) | 1.29(1.18~1.41) | 0.000 | 30.9% | 0.144 | 100% |
|  | CC+CT *vs.* TT | Asian | Random | 864/1124(2) | 1.94(1.16~3.24) | 0.011 | 86.6% | 0.006 | 100% |
|  | CC *vs.* CT+TT | Overall | Random | 15440/20522(14) | 1.23(1.14~1.32) | 0.000 | 38.2% | 0.072 | 100% |
|  | (Recessive model) | T1D | Random | 11528/14340(6) | 1.26(1.09~1.45) | 0.001 | 69.3% | 0.006 | 100% |
|  | CC *vs.* CT+TT | SLE | fixed | 2113/3827(5) | 1.18 (1.05~1.33) | 0.004 | 0.0% | 0.568 | 100% |
|  | CC *vs.* CT+TT | MS | fixed | 1679/1666(2) | 1.23(1.07~1.41) | 0.003 | 19.4% | 0.265 | 100% |
|  | CC *vs.* CT+TT | AAD | Random | 120/689(1) | 1.37(0.92~2.04) | >0.05 | - | - | 99.9% |
|  | CC *vs.* CT+TT | Caucasian | Random | 14576/19398(12) | 1.21(1.12~1.30) | 0.000 | 38.0% | 0.088 | 100% |
|  | CC *vs.* CT+TT | Asian | fixed | 864/1124(2) | 1.46(1.12~1.91) | 0.005 | 20.5% | 0.262 | 100% |
| rs10930046 | TT+TC *vs.* CC | Overall | fixed | 15868/27553(8) | 1.33(1.15~1.55) | 0.000 | 0.70% | 0.418 | 100% |
|  | (Dominant model) | T1D | fixed | 8338/10509(2) | 0.49(0.21~1.15) | >0.05 | 0.0% | 0.861 | 100% |
|  | TT+TC *vs.* CC | SLE | fixed | 6372/15908(5) | 1.37(1.17~1.60) | 0.000 | 0.0% | 0.935 | 100% |
|  | TT+TC *vs.* CC | MS | fixed | 963/960(1) | 1.49(1.15~1.55) | >0.05 | - | - | 100% |
|  | TT+TC *vs.* CC | Caucasian | fixed | 13943/22409(6) | 1.11(0.76~1.62) | 0.000 | 19.5% | 0.291 | 100% |
|  | TT+TC *vs.* CC | Asian | Random | 400/659(1) | 1.06(0.31~3.65) | >0.05 | - | - | 18.2% |
|  | TT+TC *vs.* CC | African^e^ | Random | 1525/4485(1) | 1.38(1.17~1.63) | 0.000 | - | - | 100% |
|  | TT *vs.* TC+CC | Overall | Random | 15868/27553(8) | 0.88(0.70~1.12) | >0.05 | 82.6% | 0.000 | 100% |
|  | (Recessive model) | T1D | fixed | 8338/10509(2) | 0.85(0.71 ~1.02) | >0.05 | 0.0% | 0.620 | 100% |
|  | TT *vs.* TC+CC | SLE | Random | 6372/15908(5) | 0.87(0.64~1.20) | >0.05 | 86.0% | 0.000 | 100% |
|  | TT *vs.* TC+CC | MS | Random | 963/960(1) | - | - | - | - | 100% |
|  | TT *vs.* TC+CC | Caucasian | fixed | 13943/22409(6) | 0.82(0.72~0.92) | 0.001 | 0.0% | 0.731 | 100% |
|  | TT *vs.* TC+CC | Asian | Random | 400/659(1) | 0.84(0.63~1.12) | >0.05 | - | - | 87.9% |
|  | TT *vs.* TC+CC | African^e^ | Random | 1525/4485(1) | 1.33(1.18 ~1.50) | 0.000 | - | - | 100% |

^a^ n means the numbers of case–control cohorts.

^b^ *P*(z test) <0.05 refuse H_0_: OR = 1 and statistically indicates a significant association.

^c^ When I^2^ < 50% and P_heterogeneity_ > 0.1, enrolled data were considered with mild heterogeneity and the fixed-effect model was applicable.

^d^ P means the power of the combined cohorts with α=0.05.

^e^ African refers African Americans.
